# A Simple Method of Interpretating the Effects of Electric Charges on the Volume Phase Transition of Thermosensitive Gels

**DOI:** 10.3390/gels4010024

**Published:** 2018-03-19

**Authors:** Hiroshi Maeda, Shigeo Sasaki, Hideya Kawasaki, Rie Kakehashi

**Affiliations:** 1 Professor Emeritus, Kyushu University, 46-6 Kashii 3-chome, Higashi-ku, Fukuoka 813-0011, Japan; 2 Department of Chemistry, Faculty of Sciences, Kyushu University, Motooka 744, Nishi-ku, Fukuoka 819-0395, Japan; 3 Faculty of Chemistry, Materials and Bioengineering, Kansai University, 3-3-35 Yamate-cho, Suita 564-8680, Japan; hkawa@kansai-u.ac.jp; 4 Osaka Research Institute of Industrial Science and Technology, 1-6-50 Morinomiya, Joto-ku, Osaka 536-8553, Japan; rie@omtri.or.jp

**Keywords:** volume phase transition, effects of electric charge, swelling of thermosensitive gels

## Abstract

Various apparently inconsistent experimental observations have been reported on the effects of electric charges on the volume phase transition of thermosensitive gels. A simple method of interpretating these results is presented.

## 1. Introduction

As the solvent in a polymer solution becomes poor, a phase separation will take place resulting in a phase equilibrium between two solutions of high and low polymer concentrations. What will happen, however, when the polymer chains are connected to form a network? This interesting question was first noticed and considered by Dušek and the possibility of phase transitions of networks has been discussed [1]. The volume phase transition (a discontinuous volume change) of gels, predicted by Dušek, was first experimentally observed by Tanaka on polyacrylamide gels in water-acetone mixtures [2]. The observed transition-like volume change was later ascribed to the effects of electric charges originating from acrylic acid groups as a result of hydrolysis of acrylamide groups. Later, Ilavsky et al. demonstrated [3,4], using copolymers (NDEA-*co*-MA) of *N,N*-diethylacrylamide (NDEA) and methacrylic acid (MA), that volume changes of non-ionic NDEA gels are continuous, while ionic (NDEA-*co*-MA) copolymer gels exhibited discrete volume changes. In this way, the idea was once prevailing that discontinuous volume changes of gels are favored by electric charges incorporated in polymer chains. In 1984, Hirokawa et al. observed the volume phase transition in a non-ionic thermosensitive poly(*N*-isopropylacrylamide) (NIPA) gels [5]. Then, effects of electric charges on the volume change of gels were examined by Hirotsu et al. [6] using copolymer gels (NIPA-*co*-AA) of NIPA and acrylic acid (AA). Their results indicated that introduction of electric charges enhanced the transition-like behavior observed for non-ionic NIPA gels. On the other hand, a study on another NIPA-copolymer gel, copolymers of NIPA and methacrylamidopropyltrimethylammonium chloride (MAPTAC), has indicated that transition-like volume changes of non-ionic NIPA gels disappear for copolymers containing MAPTAC more than 2 mol % and the volume changes of the latter gels are continuous [7]. These inconsistent results obtained on two NIPA*-co-*ionic component gels, NIPA-*co*-AA gels and NIPA-*co*-MAPTAC gels, have been puzzling. One way to reconcile the inconsistent results is a notion that AA and MAPTAC are different chemical entities and, hence, polymer-solvent and polymer-polymer interactions may differ significantly for the two NIPA copolymer gels [7]. Later, copolymer gels of NIPA with two kinds of ionic groups, one carboxyl group and the other amino group, have exhibited continuous temperature-induced volume changes as a result of the ionization of either ionic group, indicating chemically different groups have little consequences in this example [8]. Meanwhile, it has been concluded that NIPA-*co*-AA gels exhibit continuous volume changes with ionization [9,10], which is a similar behavior as NIPA-*co*-MAPTAC gels [7], but contrary to the reported behavior on the same kind of gel [6]. Thus, the results on two NIPA copolymer gels clearly indicate that introduction of electric charges changes a discontinuous volume change of NIPA gels to a continuous one [7,8,9,10]. The conclusion is further confirmed on another copolymer gel, NIPA-*co*-styrene sulfonate (SS) gel [9]. 

Summing up the experimental results described above, it is highly likely that an important mechanism is present regarding the effects of electric charges on the temperature-induced volume changes of gels, other than arising from different chemical properties of ionic groups. In this Communication we wish to propose a simple model to account for the charge effect under a highly approximate level of analysis.

It is pertinent to state here that continuous volume changes have been observed even for non-ionic NIPA gels, which suggests the volume phase transition of NIPA gels locates rather near the critical point. Additionally, we discuss only the case of thermosensitive non-ionic gels and the volume phase transition of ionic gels, in general, is completely outside the scope of this Communication. 

## 2. The Swelling Equations of Gels

The chemical potential of solvent water μ_w_ is measured from the reference state of pure water μ_w_^0^, Δμ_w_ = μ_w_ − μ_w_^0^. Then, Δμ_w_ can be written as Equation (1) in terms of temperature *T*, and the gas constant *R*, and the number of moles of the solvent water n_1_:

Δμ_w_/*RT* = ln(1 − φ) + φ + χφ^2^ + [∂G_elas_/∂n_1_ + Δμ_w_^ion^]/*RT*, (1)

Here, m G_elas_ represents the elastic free energy and Δμ_w_^ion^ represents the contribution arising from the introduction of ionic groups together with associated counterions. The volume fraction of polymer and the interaction parameter of polymer with solvent are denoted as φ and χ, respectively. We write the gel volume as *V*, that in the dry state as *V*_d_, and hence φ = *V*_d_/*V*. The gel volume *V*_0_ corresponds to the state in which polymer chains are in the unperturbed state. 

### 2.1. The Elastic Free Energy of Gels G_elas_

We denote the number of moles of active polymer chains in the whole gel as ν and ν* as ν/*V*_d_. We assume the relation φ_0_ *V*_0_ = φ*V* = φ_d_·*V*_d_, where φ_d_ and φ_0_ correspond, respectively, to the polymer volume fractions in the dry gel and in the unperturbed state gel. The linear deformation of the network α is given as follows:

α = (*V*/*V*_0_)^1/3^ = φ_0_^1/3^·φ^−1/3^, (2)

For the affine network with functionality = 4, G_elas_ is given as follows [11]:

G_elas_ = (3ν*RT*/2) (α^2^ − 1 − ln α), (3)

and:

∂G_elas_/∂n_1_ = 3ν*RT* [α− 1/(2α)] (∂α/∂n_1_) = ν·(*V*_1_/*V*_0_)·*RT* [(φ/φ_0_)^1/3^ − (1/2) (φ/φ_0_)], (4)

Here, the partial molar volume of solvent water is denoted as *V*_1_.

### 2.2. Uncharged Gels (Δμ_w_^ion^ = 0)

From Equations (1) and (4), we have the following expressions for Δμ_w_/*RT*:

Δμ_w_/*RT* = ln(1 − φ) + φ + χφ^2^ +ν(*V*_1_/*V*_0_)·[(φ/φ_0_)^1/3^ − (1/2)·(φ/φ_0_)], (5)

= ln(1 − φ) + φ + χφ^2^ + ν*·*RT*[(*V*_1_/φ_d_)]·[φ_d_^2/3^ <α^2^>_0_ φ^1/3^ − (1/2)·φ], (5’)

Here, the isotropic deformation factor <α^2^>_0_ is defined as <α^2^>_0_ = (*V*_d_/*V*_0_)^2/3^ = (φ_0_/φ_d_)^2/3^. There is a positive maximum of Δμ_w_/*RT* in the region of very small φ, due to the situation that polymer chains connected through crosslinks cannot be diluted indefinitely. The equilibrium swelling volume *V* is determined by the condition of Δμ_w_ = 0. When there are two maxima and one minimum of Δμ_w_/*RT* satisfying the conditions (Δμ_w_/*RT*)_min_ < 0 and (Δμ_w_/*RT*)_max_ > 0, the gel volume change induced by a change in χ can be discontinuous (transition-like behavior). The critical end point for the volume phase transition is given as follows:

∂(Δμ_w_/*RT*)/∂φ = 0 and ∂^2^(Δμ_w_/*RT*)/(∂φ)^2^ = 0, (6)

Dušek and Patterson have examined the critical condition in terms of Equation (5’) under the assumption that φ_d_ =1 and χ is a constant independent of φ [1]. They concluded that there is no unique set of parameter values [φ, χ, ν*, <α^2^>_0_] corresponding to the critical point.

On the other hand, increase of χ values with increasing polymer concentration has been frequently observed for solutions of linear polymers in poor solvents [11]. Erman and Flory have shown that discontinuous volume change of gels is possible if relevant φ-dependent χ parameters are taken into account [11]. Below, we assume χ parameters that linearly depend on φ:

χ = χ_1_ + χ_2_ φ, (7)

## 3. Effects of Electric Charge on the Swelling Behavior of Gels

At least, the following three effects are noted:

1. Contribution from the mixing with counterions.
2. Effects on G_elas_ (introduction of electric charges generally makes polymer chains stiffer).
3. Introduction of ionic groups is nothing more than the introduction of different chemical species into polymer chains. Accordingly, two (or more) kinds of χ parameters are required to describe the polymer-solvent interactions. When a kind of average χ parameter is introduced, it may significantly differ from that for the parent non-ionic polymer.

In the present highly approximate analyses, two possible contributions 2 and 3 above are ignored and only the counterion contribution is considered. Two frequently-observed phenomena with the introduction of electric charges are: (a) the concentrated phase (collapsed phase) becomes more unstable, which corresponds to an increase in the transition temperature in the case of NIPA gels; and (b) the increased swollen gel volume results in an enlarged volume change at the transition. 

### Osmotic Pressure of Small Ions 

The total mole number of counterions in the gel is *i*ν for the case of no added salt, where *i* denotes the number of charges per chain. Then, Δμ_w_^ion^/*RT* is given as follows in terms of the osmotic coefficient *g*:

Δμ_w_^ion^/*RT*= −gV_1_ (*i·*ν/*V*) = −g·*i*·(ν*·V*_1_ /*V*_0_)(φ/ φ_0_), (8)

It is to be noted that there are no small ions in the water outside the gel if no salt is added.

In the case of gels immersed in solutions containing a uni-univalent salt of the same counterion species, the Donnan osmotic pressure is approximately evaluated as follows by assuming the additivity rule [12] and the ideal behavior of the salt ions:

Δμ_w_^ion^/*RT* = −*g*·*i*·(ν*·V*_1_/*V*_0_) (φ/ φ_0_) − 2 *V*_1_ (*C*s − *C*s’), (9)

As evident in Equations (8) or (9), Δμ_w_^ion^ is negative as a result of the introduction of ionic groups. The negative contribution to Δμ_w_^ion^ arises not from the electric interactions among charges, but from the introduction of counterions. Combining Equations (5) and (9), we have the following final expression describing the swelling of thermosensitive ionic gels:

Δμ_w_/*RT* = ln(1 − φ) + φ + χφ^2^ + ν·(*V*_1_/*V*_0_) [(φ/φ_0_)^1/3^ − (1/2)·(φ/φ_0_)] − *g i*·(ν·*V*_1_ /*V*_0_) (φ/φ_0_) − 2*V*_1_·(*C*s − *C*s’), (10)

As described in the introduction part, various and inconsistent experimental results have been reported in relation to the effects of electric charges on the volume change of thermosensitive gels. Different behaviors with the introduction of electric charges will be classified into three groups: (a) a transition-like volume change is induced [3,4]; (b) the transition–like behavior observed on the uncharged gel is preserved [6]; or (c) the transition–like behavior observed on the uncharged gel disappears [7,8,9,10], but the transition-like behavior is restored on the addition of a salt [7,9].

A simple way of understanding these diverse behaviors (a)–(c) above will be presented below. To simplify the discussion, we calculate the swelling behaviors in terms of Equation (11), a simplified version of Equation (10), with *g* = φ_0_ = 1. In the absence of salt (*C*s = *C*s’ = 0):

Δμ_w_/*RT* = ln(1 − φ) + φ + χφ^2^ + ν(*V*_1_/*V*_0_) (φ^1/3^ − φ/2)− *i*· (ν·*V*_1_/*V*_0_)·φ. (11)

**Figure 1:**
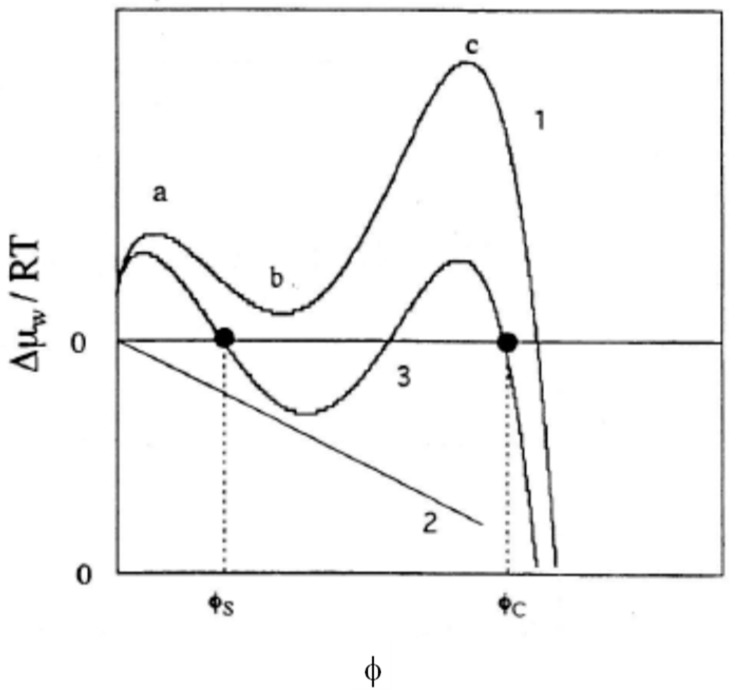
Calculated swelling curves in terms of Equation (11) in the case of the charge-induced volume phase transition. (ν *V*_1_/*V*_0_) = 0.0058. χ_1_ = 0.356 and χ_2_ = 0.840. Curve 1 (*i* = 0) shows two maxima a and c and one minimum b. Line 2 represents the last term of Equation (11) with *i* = 0.22. Two black dots in Curve 3 (*i* = 0.22) represent the volume fractions of the condensed (φ_C_) and the swollen (φ_S_) states in equilibrium with each other.

A situation corresponding to case (a) above may be indicated in Figure 1. Curve 1 in the figure represents a possible swelling curve of the uncharged gel, calculated with Equation (11) with *i* = 0. When the contribution from Δμ_w_^ion^/*RT*, the last term in Equation (11), represented with a straight line 2 is added, curve 3 results which indicates the transition-like swelling behavior.

**Figure 2:**
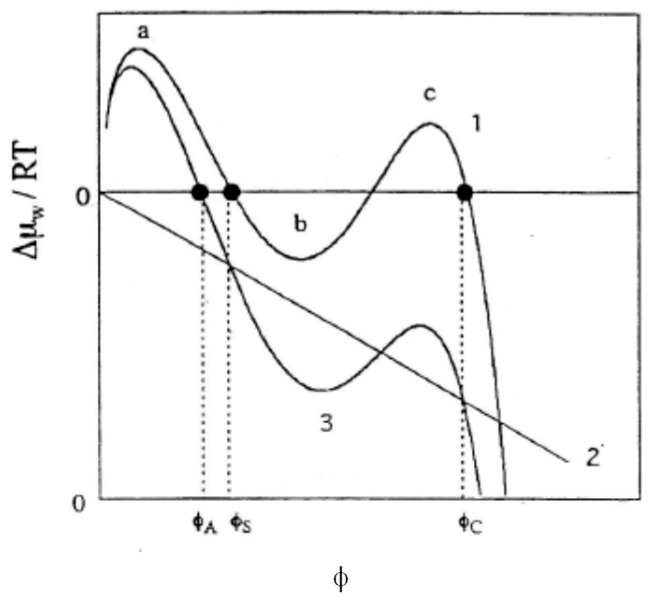
Calculated swelling curves in terms of Equation (11) in the case that the transition behavior disappears when ionic groups are introduced. (ν *V*_1_/*V*_0_) = 0.0058. χ_1_ = 0.334 and χ_2_ = 0.855. Curve 1 (*i* = 0) represents two maxima a and c and one minimum b. Two black dots represent the volume fractions of the condensed (φ_C_) and the swollen (φ_S_) states in equilibrium with each other. Line 2 represents the last term of Equation (11) with *i* = 0.005. Curve 3 (*i* = 0.22): a black dot represents the volume fractions of the equilibrium swollen volume fraction (φ_A_).

A situation corresponding to case (c) above may be indicated in Figure 2. Curve 1 in the figure represents a possible swelling curve of the uncharged gel, calculated with Equation (11) with *i* = 0, which shows a transition-like behavior between the swollen state (φ = φ_S_) and the condensed state (φ = φ_C_). When the contribution from Δμ_w_^ion^/*RT* represented with a straight line 2 is added, curve 3 results, which indicates no transition-like behavior anymore. When the contribution Δμ_w_^ion^/*RT* is small enough for the second maximum of curve 1 (denoted as c) in Figure 2 to remain positive, the transition-like behavior is preserved even in the presence of electric charges. In the case of NIPA-*co*-AA gels, a threshold of counterion osmotic pressure Π/*RT* (about 20 mM) was confirmed [9] beyond which the volume change is continuous. As far as the osmotic pressures are below the threshold, the transition-like behavior is preserved. This will be case (b) above. It is to be stated that φ-dependent χ parameters, Equation (7), are used in the calculations and prescribed values of χ_1_ and χ_2_ are chosen so that they correspond to a situation near the critical condition.

It is important, however, to examine the validity of the present model on the basis of the parameter values relevant to each reported experimental result.

A few words are in order with respect to apparently conflicting results on NIPA-*co*-AA gels [6,9,10]. An important factor affecting the swelling behavior of the gel is the pH of the media in which it is immersed. With a decrease in pH, not only the charge density of polymer chains decreases, but also the interaction parameter χ changes due probably to the hydrogen bond formation between COOH and COO^–^ groups. Different hydrations of the two groups also affect the χ parameter. Accordingly, the charge effect should be examined under the condition where carboxyl groups are nearly completely ionized and the amount of charges should be regulated not by pH, but by changing the amount of acrylic acid (AA) to be introduced. It is to be noted that a similar result as that reported in [6] was obtained in [10] at pH 5.6.

## 4. Conclusions

Disappearance of the transition-like gel volume change as a result of the introduction of electric charges could be generally understood in terms of the mechanism presented here. In the mechanism it is assumed that the mixing entropy of counterions plays a central role compared with other effects induced by ionic groups such as on the elastic properties and the χ parameter.
